# MR imaging of human brain mechanics *in vivo:* New measurements to facilitate the development of computational models of brain injury

**DOI:** 10.1007/s10439-021-02820-0

**Published:** 2021-07-01

**Authors:** PV Bayly, A Alshareef, AK Knutsen, K Upadhyay, RJ Okamoto, A Carass, JA Butman, DL Pham, JL Prince, KT Ramesh, CL Johnson

**Affiliations:** 1 Department of Mechanical Engineering and Materials Science, Washington University in St. Louis; St. Louis, MO, USA; 2 Department of Electrical and Computer Engineering, Johns Hopkins University; Baltimore, MD, USA; 3 Center for Neuroscience and Regenerative Medicine, The Henry M. Jackson Foundation for the Advancement of Military Medicine; Bethesda, MD, USA; 4 Hopkins Extreme Materials Institute, Johns Hopkins University; Baltimore, MD, USA; 5 Clinical Center, National Institutes of Health; Bethesda, MD, USA; 6 Department of Mechanical Engineering, Johns Hopkins University; Baltimore, MD, USA; 7 Department of Biomedical Engineering, University of Delaware; Newark, DE, USA

**Keywords:** traumatic brain injury, computational models, magnetic resonance imaging, deformation, strain

## Abstract

Computational models of the brain and its biomechanical response to skull accelerations are important tools for understanding and predicting traumatic brain injuries (TBIs). However, most models have been developed using experimental data collected on animal models and cadaveric specimens, both of which differ from the living human brain. Here we describe efforts to noninvasively measure the biomechanical response of the human brain with MRI—at non-injurious strain levels—and generate data that can be used to develop, calibrate, and evaluate computational brain biomechanics models. Specifically, this paper reports on a project supported by the National Institute of Neurological Disorders and Stroke to comprehensively image brain anatomy and geometry, mechanical properties, and brain deformations that arise from impulsive and harmonic skull loadings. The outcome of this work will be a publicly available dataset (http://www.nitrc.org/projects/bbir) that includes measurements on both males and females across an age range from adolescence to older adulthood. This article describes the rationale and approach for this study, the data available, and how these data may be used to develop new computational models and augment existing approaches; it will serve as a reference to researchers interested in using these data.

## 1. Introduction

### 1.1. Scope and Purpose

Traumatic brain injury (TBI), including mild TBI and concussion, is widespread^65,95^ and can have lasting and severe consequences^10,63^. Large, fast deformations of brain tissue caused by high skull accelerations undoubtedly contribute to TBI pathology^43,105,110^, and repeated, smaller brain deformations caused by sub-concussive accelerations likely underlie features of chronic traumatic encephalopathy (CTE)^10,110^. However, the relationships between skull acceleration, brain deformation, pathophysiology, and brain function are not fully understood. These biomechanical relationships are critical to unraveling the mechanisms of TBI and CTE and ultimately improving approaches to prevention and treatment. For instance, knowing how much acceleration and deformation leads to injury and what regions of the brain are likely to undergo injurious deformations can inform helmet design^91^. However, it is ethically impossible to answer these questions by experimental studies that risk injury to human subjects.

Computer simulation of human skull and brain mechanics offers an appealing and potentially powerful approach to provide both qualitative and quantitative insights into TBI^51^. A computational model can simulate the brain’s response to skull accelerations of arbitrary magnitude and direction, providing predictions of 3D displacement vector fields and strain tensor fields with high spatial and temporal resolution throughout the entire brain. **However, it is crucial to remember that the predictions of computational models are not measurements**. The accuracy and utility of these predictions depends on many factors, including the assumptions and approximations used to build the model^38,69^, but most critically on the data used in model development. To create an accurate and useful model, parameters such as the mechanical properties of brain tissue and its connections to the skull must be obtained from experimental measurements^76^. Similarly, the accuracy of *predicted* deformations can only be truly assessed by quantitative comparison to corresponding *measurements* of deformation (strain).

**Figure 1.**
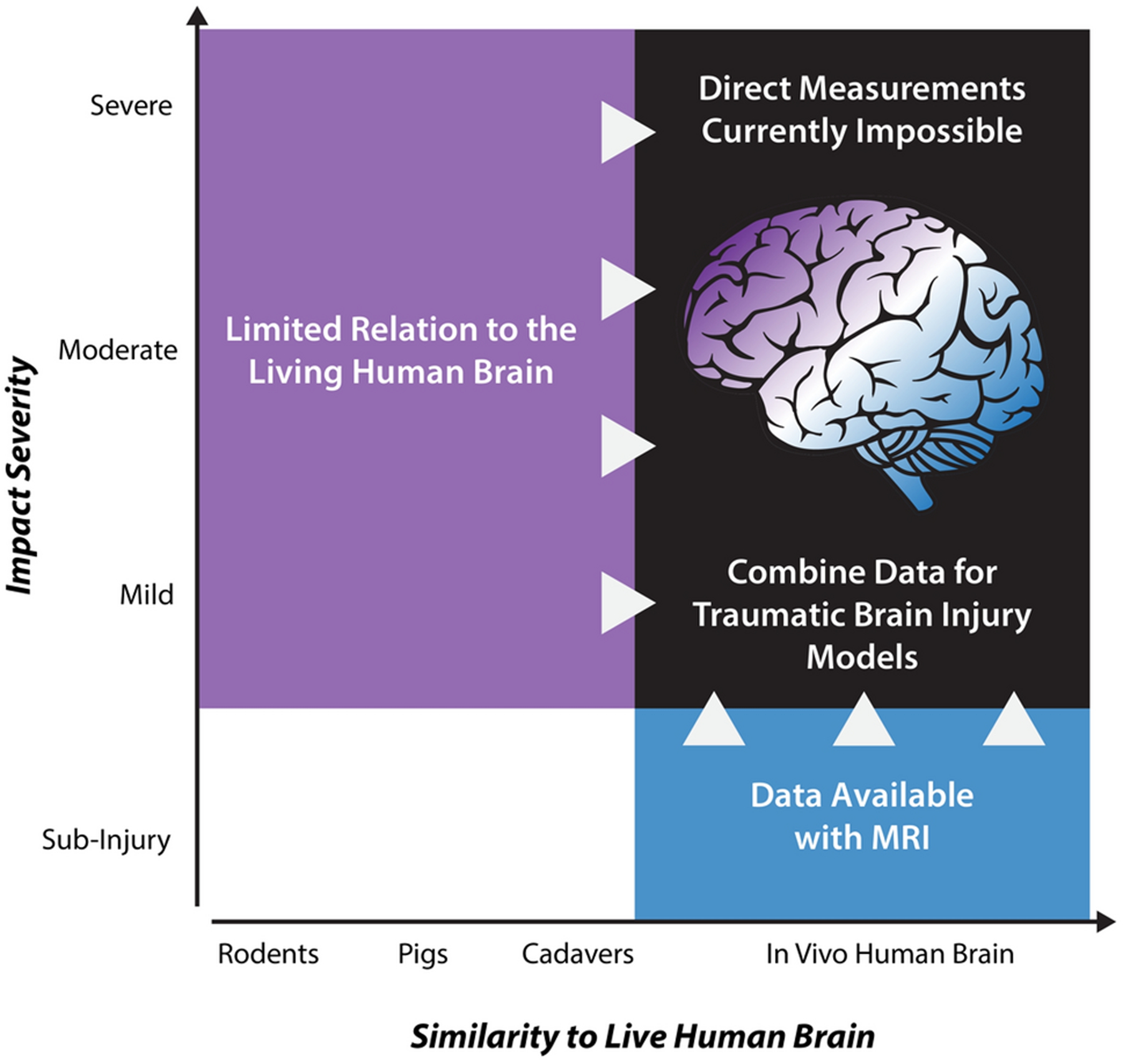
Computational models of traumatic brain injury (TBI) require experimental measurements of brain deformation in response to skull accelerations. Most models are built from data obtained in model systems (cells, animals, or cadaveric specimens) at high strains and strain rates relevant to TBI. However, none of these systems completely recapitulates the behavior of the living human brain. We propose to augment these data with noninvasive MRI measures of brain deformation at safe strains and strain rates to develop, calibrate, and evaluate TBI models.

Given the present inability to capture experimental data on the living human brain at high (injurious) strains and strain rates, researchers have instead employed alternative methods to study skull-brain mechanics using animal models^21,109^ and cadaveric specimens^1,2,45,46^. Unfortunately, both the animal and cadaver brains differ from the intact, living human brain, thus limiting the utility of these data *alone* for developing models of TBI. Instead, we propose that noninvasive MRI measurements of deformation of the living human brain can be used to augment the development, calibration, and evaluation of computational models of brain biomechanics (Figure 1).

To that end, we report new data on the mechanical behavior of the human brain, obtained using MRI techniques in human volunteers during sub-injury levels of skull acceleration, and made publicly available with support from the National Institute of Neurological Disorders and Stroke (NINDS) of the National Institutes of Health (NIH). We aim to provide comprehensive measurements of 3D deformation of the human brain through (i) measurements of motion in response to impulsive or harmonic skull motion, (ii) material properties, (iii) anatomy, and (iv) microstructure of the human brain. These measurements capture key features of the response of the intact, living human brain to skull motion and complement data available from cadaveric and animal studies. The major outcome of the project will be a publicly available dataset (http://www.nitrc.org/projects/bbir) accompanied by tools for using these data to parameterize and evaluate computational models of brain biomechanics under sub-injurious loading conditions.

### 1.2. Computational TBI Models

Computational models of the brain have become a valuable tool in biomechanics research to investigate TBI mechanisms, predict injury risk, and develop safety gear^51,55,111^. Models can illuminate the biomechanics of the brain during an impact by applying potentially injurious kinematic conditions that are impossible to achieve with human subjects, or even cadaveric specimens. These models have been used to create injury risk curves through proposed summary metrics of deformation, such as maximum principal strain (MPS), maximum axonal strain (MAS), and the cumulative strain damage measurement (CSDM)^30,60,111,122^. These risk curves, in turn, have informed injury mitigation approaches and consumer safety standards. The convenience and wide use of brain computational models emphasize the critical need for assessment of their biofidelity.

To draw physiologically relevant conclusions about TBI risk and mitigation, a model’s biomechanical response should ideally resemble that of a live human brain. However, few computational brain models have been evaluated by comparison to experimental measurements of living human brain deformation^81,124^. Instead, modelers have largely relied on cadaveric or animal studies. Many brain models have been evaluated using a single set of data from cadaveric brain motion under blunt impact^45,46^; more recently, measurements of high-rate, rotational cadaveric brain motion have been obtained using sonomicrometry^1,2^. Some models have also leveraged data from animal studies^44,111^, reconstructed impacts^23,102,116^, and on-field measurements in contact sports^30,31^, though none of these data directly provide the necessary measurements of brain deformation to properly develop or calibrate a model for the *in vivo* human brain.

The evaluation and interpretation of computational brain models is confounded by a number of factors. Model predictions depend on: (i) physical parameters like material properties, applied loading, and boundary and interface conditions; (ii) numerical parameters like mesh element type and size, time step, and temporal integration scheme; and (iii) geometry and anatomy, which may differ between the model and an individual subject^38,69^. Model outputs, usually displacement or strain time-history throughout the brain, are also reduced to a single global strain measure (e.g., MPS) thus ignoring the richness of spatial and temporal variations which could be used to evaluate a given model.

Detailed anatomy, microstructure, and strain measures from individual subjects can be used to create subject-specific models that account for variations in head and brain morphometry. Incorporating subject-specific anatomy has been motivated by studies showing variability in predicted brain deformation due to variations in geometry^66^, axonal tract alignment^36^, and impact kinematics^117^. While many models have recreated patient-specific impact scenarios, only a few studies have evaluated models using individual subject anatomy^31,55,66,68,80^ and strain data^124^. Additionally, subject-specific material properties, such as those derived from magnetic resonance elastography (MRE), have the potential to improve models though this has been explored only recently^37,70^.

### 1.3. Imaging Brain Biomechanics with MRI

MRI is well-suited to elucidate the mechanics of the live, human brain. Structural MRI offers excellent contrast in soft tissues, which enables high spatial resolution of subject-specific brain anatomy that can be segmented and classified using automated algorithms^22,27,52^. Diffusion tensor imaging (DTI) provides information about the orientation of white matter fibers^9,82^. Most importantly, there are several well-developed MRI acquisition techniques that are sensitive to motion and can be used to image how the brain moves in different conditions^88,106,112^. These methods offer opportunities for examining brain motion in ways that illuminate traumatic events, but at safe displacement and strain levels, using both harmonic and impulsive loading of the skull.

Magnetic resonance elastography (MRE) is a phase-contrast MRI technique that is sensitive to harmonic motion at scales of hundreds of nanometers to microns^71,85^. In MRE, vibration by an external driver leads to shear waves in soft tissue and the speed of shear waves is used to estimate tissue mechanical properties. MRE has been used to study the effects of neurodegeneration on brain viscoelasticity^48,83^ (the diseased brain is significantly softer than the healthy brain) as well to study normal development^58,78,119^ and aging^4,49,100^. Notable differences in tissue properties have been observed across the lifespan and between individuals^47,50^. Outcomes from MRE include whole-brain maps with spatially resolved tissue stiffness and viscosity, which are valuable data to incorporate into subject-specific TBI models. Additionally, the computed displacement fields illustrate the brain response to harmonic loading, including how waves propagate through the brain^20,107^ and generate different regional strain patterns^86^. Recent efforts have also sought to measure relative motion between the skull and brain to characterize their coupling^6,7,120^.

The brain’s response to impulsive loading of the skull (as in blunt impact) can be imaged at sub-injury levels using tagged MRI, an imaging method originally developed to noninvasively measure contractile function of the heart^5,121^. In tagged MRI, the longitudinal magnetization in the tissue is modulated to create temporary patterns of contrast that move with the tissue and can be analyzed to create maps of deformation and strain^67,87,118^. Advances in imaging and analysis techniques have established the ability to track 3D strains over time throughout the brain^41^. In the current method, repeated impulsive loading is applied to the skull through custom devices that restrict head motion to generate a repeatable accelerative loading on the order of 2–3 g^11,25^ (20–30 m/s^2^) or 150–350 rad/s^2 17,61^. These loading conditions are comparable to or smaller than accelerations due to other non-injurious, everyday activities^29^. This loading has been applied in several directions, including neck rotation^17,41,61,62,98^ (rotation in the axial plane), neck extension^11,41,61^ (translation and rotation in the sagittal plane), and neck flexion^25^. Diverse loading scenarios can uncover potential similarities and differences in brain response from impacts in different locations and directions.

Importantly, computational models that predict injury based on strain should be evaluated by comparison to experimental strain measurements^124^. The full-field measurements of dynamic, 3D strain throughout the brain in vivo at sub-injury levels provided by MRI-based methods are well-suited to this purpose, and complement estimates of strain from relatively sparse marker sets in the cadaveric brain^125,126^ at higher loading levels.

### 1.4. Study Design & Overview

The goal of this paper is to provide an overview and guide to new data describing the biomechanics of the living human brain. These new imaging and brain deformation datasets will help address gaps in the development and evaluation of computational TBI models. These data reflect spatially-resolved, three-dimensional motion from the whole brain in living humans. They will enable model construction and parameterization with anatomy (from MRI), microstructure (from DTI), and material properties (from MRE), and evaluation by comparison to subject-specific brain deformation (from tagged MRI). Data sets from multiple subjects will be collected and released to enable characterization of variability in the biomechanical response.

Data are being collected at three different sites: Washington University in St. Louis, University of Delaware, and the Clinical Center at the National Institutes of Health. The scan protocols differ between sites and focus on different aspects of brain biomechanics. Procedures include tagged MRI to measure brain response to impulsive loading, MRE to measure brain response to harmonic motion at multiple frequencies, and high-resolution MRE to map brain material properties. In the following section, we describe the measurements that will be shared with the community, and the methods used to obtain these data. We then discuss considerations and challenges in developing, calibrating, and evaluating TBI models using the full field data from this study.

## 2. MRI-Based Measurements and Analysis

Participants in this study will complete a scanning session that includes one of tagged MRI of impulsive loading, MRE of harmonic loading, or high-resolution MRE for material property mapping, depending on the site where data is collected, along with associated anatomical images. Each site will acquire data on approximately 100 participants including both males and females across a large age range (14–80 years old, split into four groups) to capture relevant age and sex differences. The complete dataset will include approximately 300 participants with data to be uploaded as it is collected.

### 2.1. Brain Response to Impulsive Loading Measured by Tagged MRI

Data on how the brain deforms in response to impulsive loading are acquired at the Clinical Center of the National Institutes of Health (NIH). These studies use tagged MRI to generate measurements of 3D deformation throughout the entire brain in response to repeated skull loading at sub-injury acceleration levels^41,61^.

We use the harmonic phase finite element method (HARP-FE) to compute 3D displacements and strain fields^41^ from multi-slice tagged MRI acquired during impulsive loading of the skull. We have previously demonstrated that this approach provides accurate and reliable 3D measurements of brain deformation^41,61^. The motions of interest are neck rotation (rotation within the axial plane of the head) and neck extension (rotation within the sagittal plane of the head). MRI-compatible devices constrain motion of the volunteer’s head to generate repeatable, mild head accelerations of approximately 150–350 rad/s^2^ and angular velocities at impact of roughly 1.5–3.5 rad/s, which are well below injury-level conditions. An angular position sensor provides measurements of angular position, velocity, and acceleration of the head cradle during impact. Brain motion is imaged with a multi-slice acquisition that incorporates tags along three orthogonal directions to provide full brain coverage at 18 ms temporal resolution, using fewer than 150 repetitions^61^. All tagged MRI data at NIH is acquired on a Siemens 3T Biograph scanner with flexible array and spine receive coils used together to accommodate the head support device.

**Figure 2.**
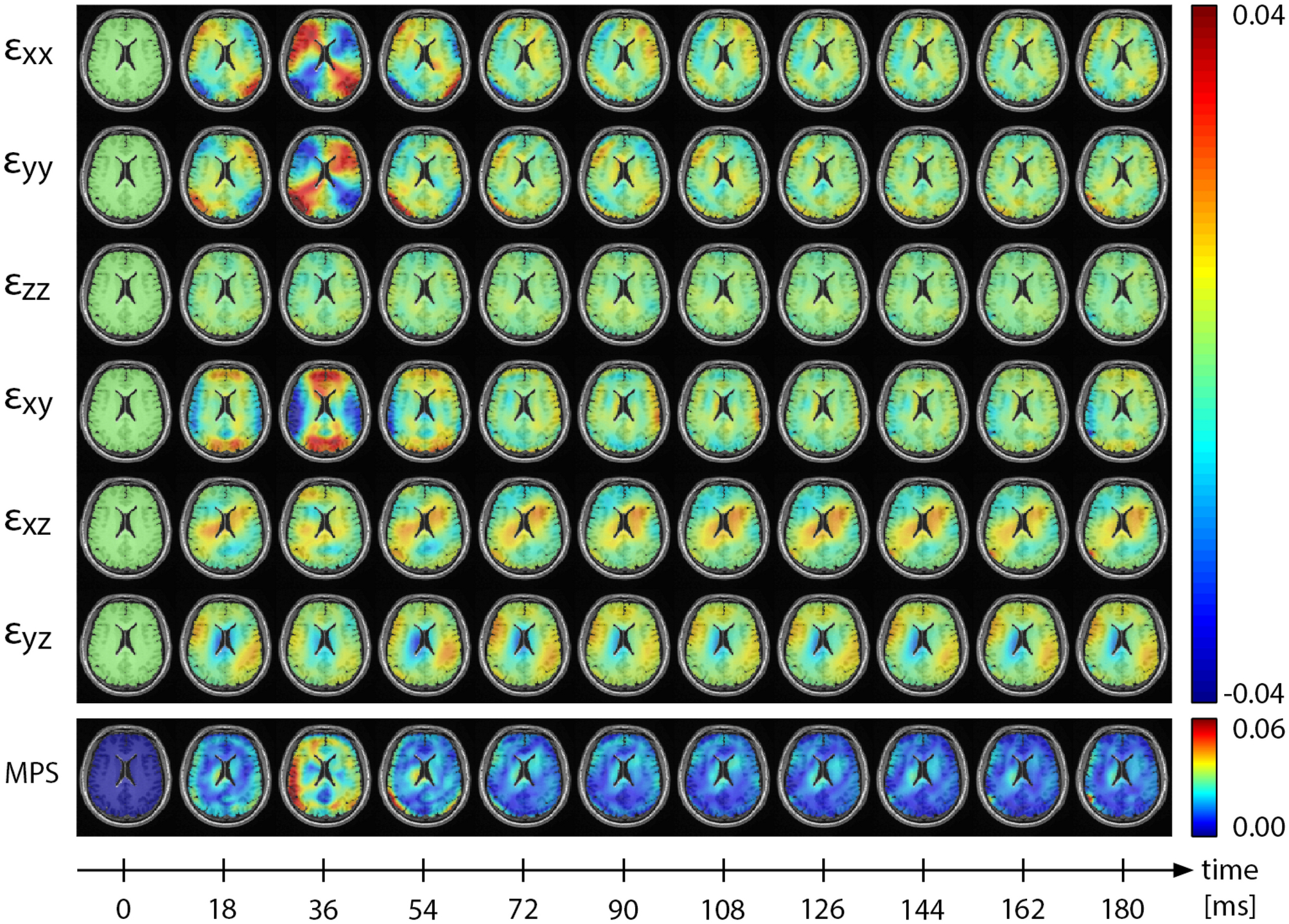
Deformation (strain) in the brain of a human volunteer, measured with tagged MRI during its response to impulsive angular acceleration of the head. All components of the strain tensor (*ε*_*ij*_) are captured throughout the entire brain and across time. These strain fields can be used to calculate summary strain metrics, such as maximum principal strain (MPS).

Following MRI acquisition, 3D motion tracking is performed using the HARP-FE method, which uses HARP images as a forcing function for a finite element mesh^41^. HARP-FE generates dense, 3D measurements of Lagrangian displacement that are then projected from the finite element mesh back to the image space for the calculation of Lagrangian strains^61^. The outcomes of the tagged MRI experiments are full vector, time resolved measures of strain at each point in the brain for the entire duration of sampling around the impact (Figure 2). From these data many summary outcomes describing strain response can be computed, and anatomical and diffusion-weighted images can be combined with these measurements to estimate axonal strain and to map deformation of brain structures and vasculature^61^. In a previous study with the same acquisition and impact device, measured MPS values (95^th^ percentile) in the brain ranged from 0.019 to 0.053^61^.

### 2.2. Brain Response to Harmonic Skull Motion Measured by MR Elastography

Data on the brain response to harmonic motion are acquired at Washington University in St. Louis (WUSTL) using MRE over a wide range of frequencies. MRE offers a robust technique to capture full vector, 3D displacement fields throughout the entire brain^48^. The human brain appears to exhibit preferred modes of oscillation which may be selectively excited at different frequencies^24,64^, and thus these studies complement tagged MRI studies, in which broadband excitation is provided by impulsive loading. We additionally seek to capture both skull motion and brain motion^6,7,120^ to illuminate features of the skull-brain interface relevant to TBI.

Harmonic skull displacement is generated by the Resoundant pneumatic actuator system (Resoundant, Rochester, MN) with one of two passive drivers in contact with the head: a soft pillow driver for occipital actuation^84^, as is commonly used in brain MRE, or a flexible silicone bottle for lateral actuation, developed more recently for multi-excitation MRE experiments^3,107^. The WUSTL MRE protocol includes five separate scans with either occipital or lateral actuation at 20, 30, 50, 70, and 90 Hz. MRE displacement data is acquired with an echoplanar imaging (EPI) sequence for robust, whole-brain displacement data across a range of frequencies. Motion-encoding gradients (MEGs) applied in the sequence are tailored for frequency to balance motion sensitivity with echo time through fractional encoding^97^ or additional gradients. At each frequency, we acquire MRE displacement data over the whole brain (240×240×132 mm^3^) with 3 mm isotropic voxel resolution and 4 time points per period. Immediately following the whole brain acquisition, we use the same EPI sequence to acquire MRE displacement data with reduced MEG strength on six slices (3 mm isotropic voxels, 18 mm slice spacing) and 8 time points per period. This data captures rigid-body motion with minimal or no phase wrapping and allows us to reliably temporally unwrap the whole brain phase data^6^. Acquisition time for both scans at each frequency range from 5 minutes (20 Hz) to 3 minutes (90 Hz). The remainder of the protocol includes anatomical and diffusion-weighted scans, as described below. All imaging data at WUSTL are acquired on a Siemens 3T Prisma scanner with a 20-channel head/neck coil.

**Figure 3.**
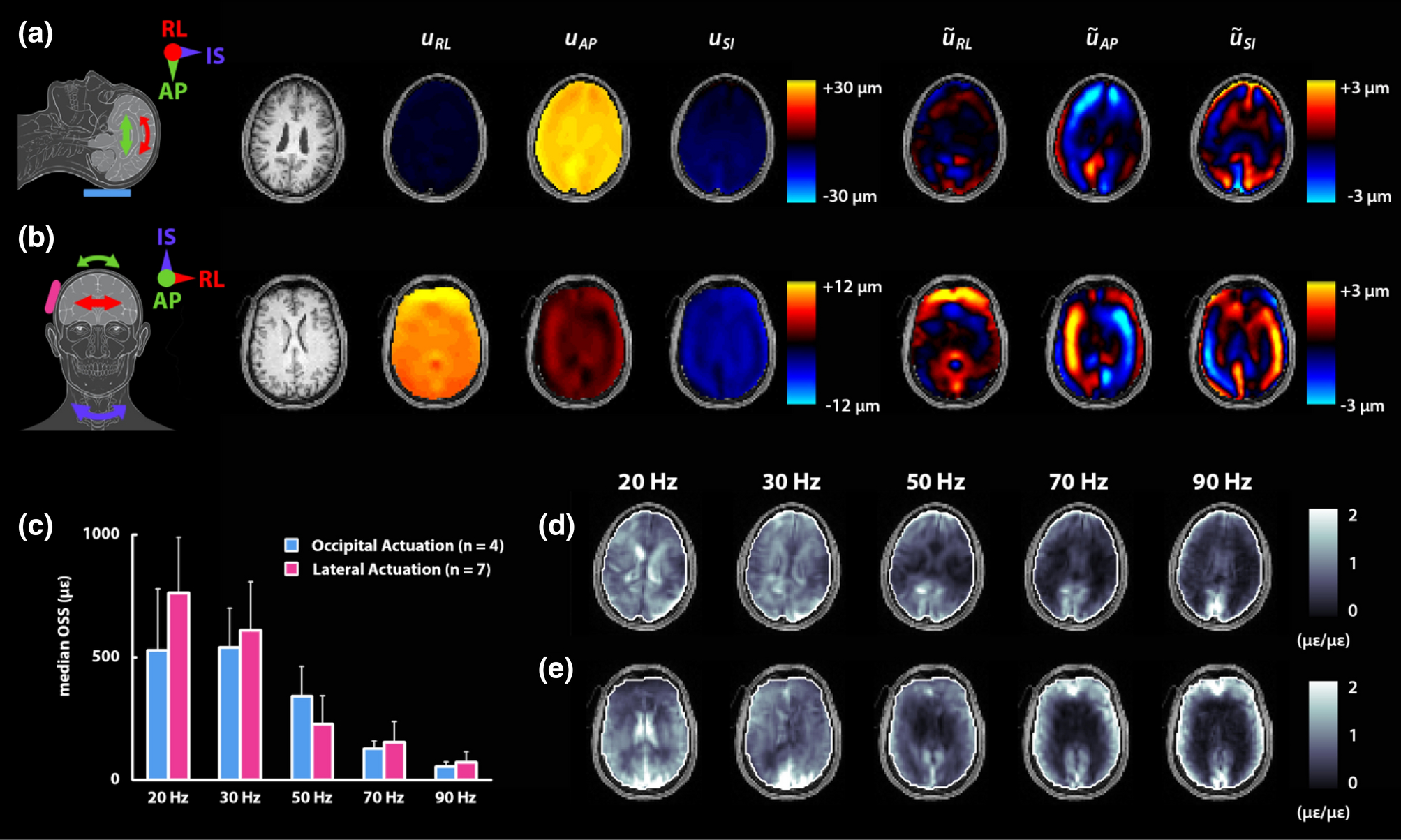
Illustration of brain response to applied harmonic motion from MRE: Total displacement (*u*) and dynamic deformation (“wave displacement”, $\tilde{u}$, obtained by subtracting rigid-body motion from *u*) of the brain with (A) occipital actuation and (B) lateral actuation at 50 Hz. Note the difference in scale for total displacement between actuation types. (C) Median octahedral shear strain (OSS) in the brain as a function of actuation frequency. Error bars show standard deviation of median values between subjects (N=4, occipital; N=7, lateral). Normalized OSS distributions, calculated as OSS divided by median OSS in the brain, for (D) occipital actuation and (E) lateral actuation.

MRE images are masked to isolate voxels in the brain and the skull and scalp. From the reduced MEG strength displacement data, we estimate the rigid-body motion of the brain and scalp^7^, which we then use to temporally unwrap the whole-brain phase field to obtain accurate full-field displacement data (3D displacement vector at each voxel)^6^. Displacement data are processed to obtain rigid-body motion and dynamic deformation, including a full 3D strain tensor in each brain voxel at each frequency. These field variables are used to calculate scalar and vector measures that characterize the frequency response of the brain to applied harmonic motion^86^ (Figure 3), such as wave propagation direction^20^, octahedral shear strain^75^, and axonal strain by incorporating fiber direction from DTI data.

### 2.3. Brain Material Properties Measured by MR Elastography

**Figure 4.**
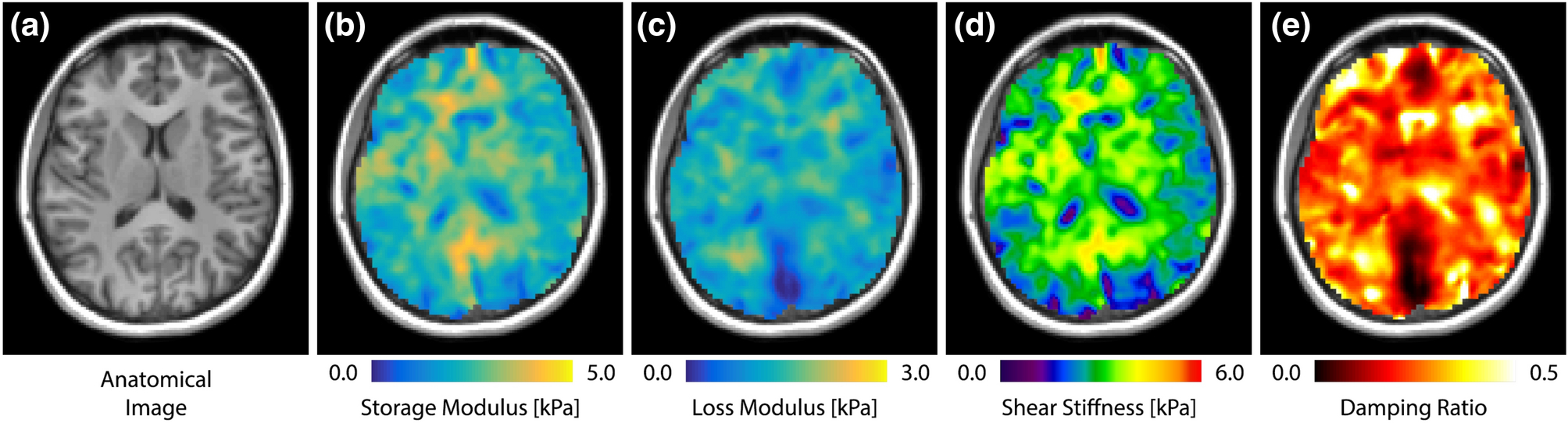
Illustration of mechanical property map outcomes from high-resolution MRE: (A) Corresponding anatomical image; (B) shear storage modulus, *G’*; (C) shear loss modulus, *G”*; (D) shear stiffness, *μ*; and (E) damping ratio, *ξ*. Property maps are created for each of three frequencies separately, 30, 50, and 70 Hz (only 50 Hz shown).

Measurements of the mechanical properties of the human brain are performed at the University of Delaware (UD). At UD, we use a high-resolution MRE sequence to capture whole-brain displacement data and an advanced inversion algorithm to generate spatially resolved maps of complex shear modulus, stiffness, and damping ratio (Figure 4). We have previously demonstrated that these methods result in property maps with improved resolution and reliability^57^, which can be used for subject-specific modeling with heterogeneous properties. As brain tissue mechanical properties are frequency-dependent^19,99,113^, capturing this behavior is critical for accurate and appropriate modeling, and thus MRE exams are performed at multiple frequencies.

MRE displacement data are acquired with our 3D multiband, multishot spiral sequence^56^. This sequence allows for high spatial resolution through data sampling designed to maximize signal-to-noise ratio efficiency and minimize artifacts, with further correction for field inhomogeneities and motion-induced phase errors during image reconstruction in PowerGrid^16^. The MRE protocol includes three separate scans at 30, 50, and 70 Hz, with 1.5 mm isotropic imaging resolution and whole-brain coverage (240×240×120 mm^3^). To reduce scan time, we also implemented the OSCILLATE encoding scheme^77^. OSCILLATE exploits spatiotemporal correlations in MRE to reconstruct a set of images with reduced rank from undersampled data. This acceleration results in acquisition times of approximately 5 minutes per frequency. MRE scans encode displacements generated by the Resoundant pneumatic actuator system with a soft pillow driver. The remainder of the protocol includes reduced MEG scans to measure rigid body motion, as described above, and anatomical and diffusion-weighted scans, as described below. All imaging data at UD are being acquired on a Siemens 3T Prisma scanner with a 64-channel head/neck coil.

From each imaged displacement field, we estimate brain tissue material properties using the nonlinear inversion algorithm (NLI)^74^. NLI is a finite element-based optimization approach to the MRE inverse problem that models tissue as a heterogeneous, viscoelastic solid. NLI estimates the complex shear modulus G* = *G’*+*iG”*, with the storage modulus *G’* describing elastic behavior of tissue and the loss modulus *G”* describing the viscous behavior. Additionally, we calculate the shear stiffness *μ* = 2|G*|^2^/(|G*|+*G’*), which generally describes the resistance of a viscoelastic material to harmonic forcing and is related to the square of the wave speed^71^, as well as the damping ratio *ξ* = *G”*/2*G’*, which describes the relative viscous-to-elastic behavior of the material^72^. These parameters are all determined at a single frequency and thus are reported for each of 30, 50, and 70 Hz; however, opportunity exists to model the frequency-dependence of the shear modulus directly in the NLI formulation^113^.

### 2.4. Brain Geometry and Microstructure from Anatomical and Diffusion-Weighted MRI

In addition to probing the mechanical behavior of the brain, MRI is used to characterize its anatomical structure, thus enabling construction of detailed brain models. MRI sequences can be tailored to map soft tissue structure, axonal orientation, and vasculature. T_1_- and T_2_-weighted images are acquired at approximately 1 mm isotropic spatial resolution as part of the protocol at each site. T_1_-weighted imaging provides exquisite contrast between gray and white matter, while T_2_-weighted imaging offers superior definition of the subarachnoid space, a key component in defining accurate boundary conditions in computational brain injury models. Diffusion tensor imaging (DTI) data are acquired using at least 30 non-collinear gradient directions at approximately 2 mm isotropic spatial resolution to characterize white matter fiber tracts. Finally, both susceptibility-weighted imaging (SWI) and time-of-flight MR angiography (MRA) are acquired in a subset of participants. These two acquisitions offer complementary depictions of cerebral vasculature, with MRA more revealing of arteries and SWI more sensitive to veins. Because of its ability to achieve submillimeter spatial resolution within a reasonable acquisition time^103^, SWI is also useful in finding thin dural membranes such as the falx cerebri and tentorium cerebelli.

**Figure 5.**
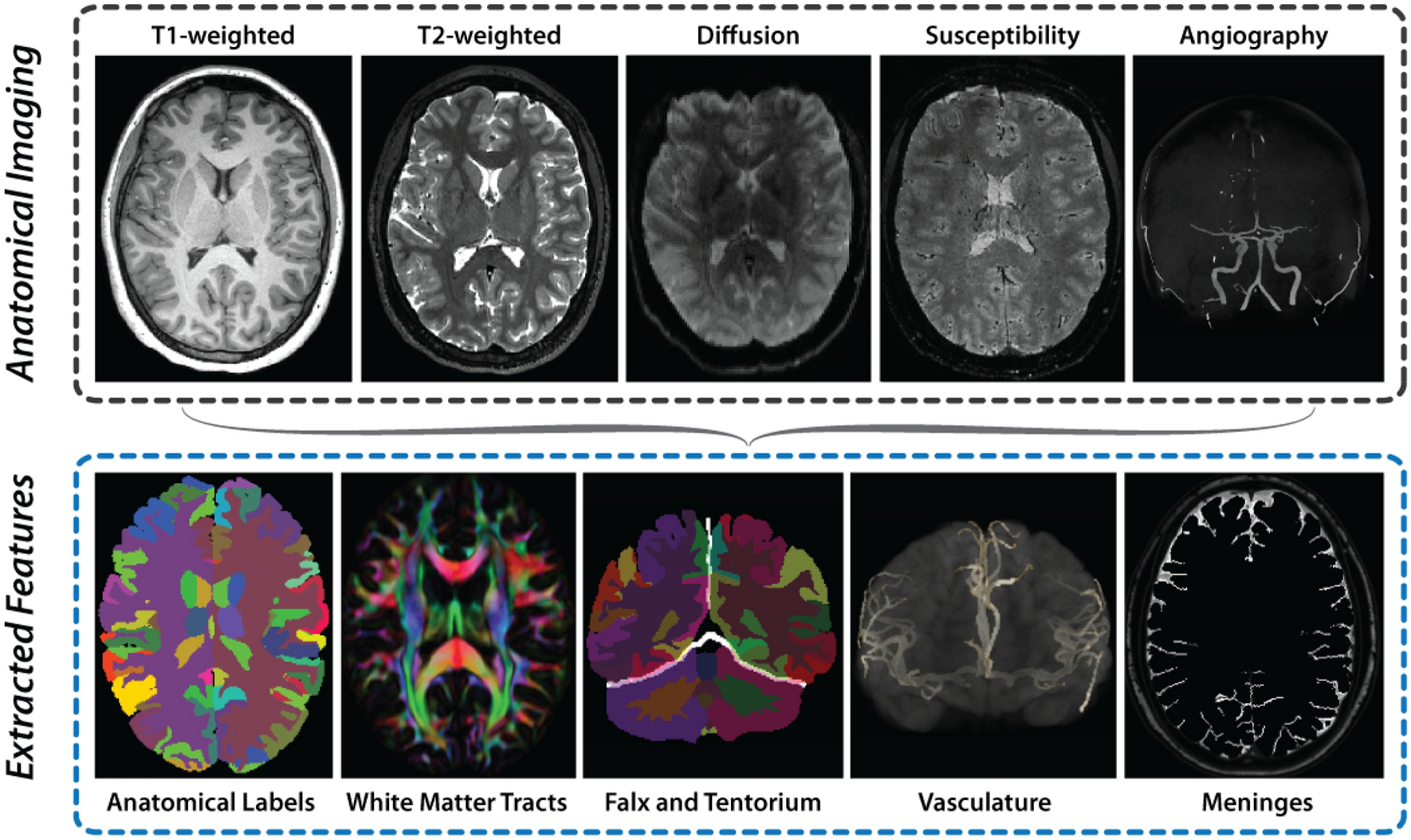
Images acquired to extract brain geometry and microstructure features relevant to computational brain models. Imaging data includes T_1_- and T_2_-weighted imaging, diffusion tensor imaging, susceptibility-weighted imaging, and time-of-flight MR angiography. From these data we extract labels of anatomical regions, white matter tract orientation, location of the falx and tentorium dural membranes, and the location of vasculature and meninges. Imaging data and extracted features are made available along with corresponding biomechanical imaging data.

Images are processed to provide voxel-wise label maps of various anatomical structures as well as to measure diffusion properties in white matter (Figure 5). To label cortical and subcortical gray matter structures, T_1_- and T_2_-weighted images will be skull-stripped^96^ and processed with a multi-atlas segmentation and cortical reconstruction algorithm^52^. From these same images and the segmentation labels, the subarachnoid space can be automatically defined, as preliminarily described by Glaister, et al^40^. Both the falx and tentorium will be reconstructed using a fast-marching, multi-atlas-based segmentation based on T_1_-weighted and SWI images^39^. Diffusion-weighted images will be processed using the TORTOISE software package^53,90^, leading to estimates of the diffusion tensor, fractional anisotropy, and mean diffusivity at every voxel, with major white matter fiber tracts labeled automatically^12^. Tools for automatically defining vasculature from MRA images are currently in development, with initial results described by Bilgel, et al^14^. This processing pipeline is executed on each dataset collected according to the exact protocol from each site, with original and processed images shared along with biomechanics imaging data.

### 2.5. Data Sharing

To facilitate the dissemination of the data and promote the sharing of results we have created a website hosted on the Neuroimaging Tools and Resources Collaboratory (NITRC) titled “Brain Biomechanics Imaging Resources” (http://www.nitrc.org/projects/bbir). The site lists the available datasets to download and Matlab software to facilitate initial exploration of the data. There are two corpuses of data: tagged MRI and MRE. The tagged MRI data to be shared consists of: 1) estimated displacement fields; 2) calculated strain fields; 3) head kinematics (angular positions, velocity, acceleration); and 4) corresponding anatomical MR images. The MRE data to be shared consists of: 1) raw displacement fields; 2) calculated strain fields; 3) estimated shear modulus maps; 4) head acceleration (if recorded); and 5) corresponding anatomical MR images. MRE and tagged MRI data will be shared in their original native image space, and the processed anatomical data will be registered to a standardized space (MNI-152^28^) and resampled at 0.8 mm isotropic digital resolution prior to sharing. For both the tagged MRI and MRE subjects, demographic and anthropometric information—e.g., age, sex, height, and weight—is provided. The data are currently distributed in the Neuroimaging Informatics Technology Initiative (NIfTI) file format. NIfTI is an open file format that provides a standardized way to disseminate the data without custom software libraries. All datasets are released under GNU general Public License v3.0 and with the acceptance of the NITRC copyright and license terms. All data collected in this study is collected following procedures approved by the appropriate Institutional Review Board, and all participants provide written, informed consent that included the public sharing of de-identified data.

Data acquisition is ongoing, so the number of subjects and available data will increase. Additionally, as processing pipelines enter stable phases, additional processed results will be made available on the data repository site. Furthermore, we anticipate that as these pipelines undergo revisions, there may be multiple updates to the available processed data.

## 3. Using Imaging Data to Improve Computational Models of Brain Biomechanics

**Figure 6.**
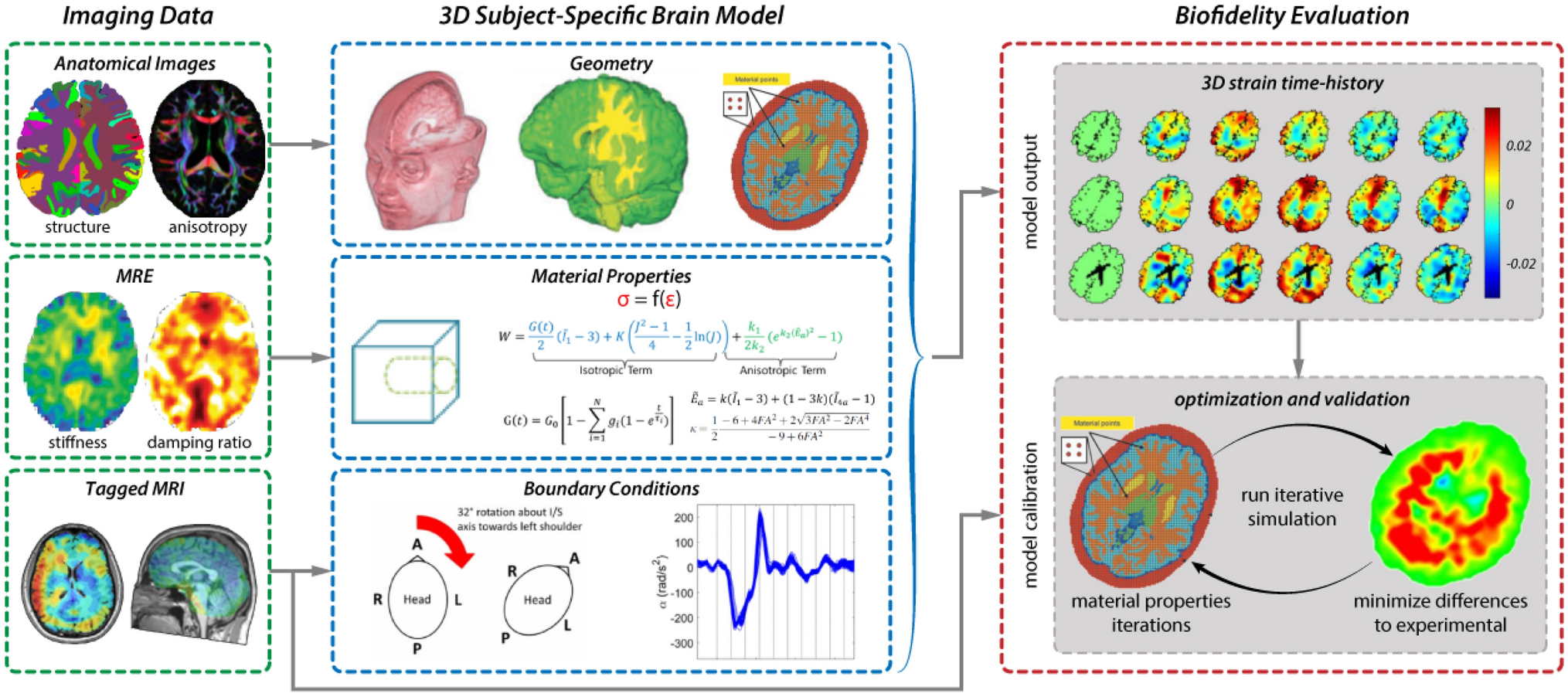
Illustration of an example workflow for developing a computational brain biomechanics model from MRI data. Shown schematically from left to right, model development requires: (i) parameterization with anatomy, microstructure, material properties, boundary conditions, and input kinematics; (ii) calibration by comparison to a subset of measurements; and (iii) evaluation by comparison to different measurements.

The data described in this work and made publicly available through this project can contribute to the *development*, *calibration*, and *evaluation* (or *validation*) of new brain biomechanics models, as illustrated by Figure 6, which, in turn, can be used to make engineering *predictions* about brain deformation in TBI. Even with the availability of such a comprehensive dataset, building an ideal computational model is challenging and requires the development of tools to appropriately extract information relevant to brain injury, as well as the careful consideration of prediction uncertainty.

### 3.1. Perspectives on the Next Generation of Computational Models

We envision that the next generation of computational models of brain biomechanics will comprise a large ensemble of subject-specific models, spanning a wide range of ages and both sexes, as well as population-average models for groups of different age and sex; these models would be developed, calibrated, and quantitatively evaluated by comparison to a wide range of high-quality experimental data.

Creating models based on subject-specific imaging data can overcome a significant source of uncertainty in model development and model performance: the anthropometric, biomechanical, and physiological differences between subjects which may affect the brain’s mechanical response. A model of a single subject also provides greater insights into model biofidelity, as well as an individualized prediction of TBI risk. An ideal scenario for model evaluation would be to build subject-specific computational models using geometry from anatomical images of each subject, and then to compare the output of these models with experimental brain deformation data for that subject. Each model would be calibrated using data from the individual subject under certain loading conditions, before predicting subject brain deformation under other loading conditions. *To this end, a major contribution of this study is to provide datasets for creating and evaluating these models*. We note that not every subject will complete every biomechanics imaging scan, and thus only a subset of subject-specific models will be built using *all* classes of available data. Specifically, we expect that fewer than 5 subjects will undergo both tagged MRI studies, which provide response to impact and thus data for model calibration, and MRE studies, which provide subject-specific material properties. Most subject-specific models will need to be built using individual anatomy and group-average material properties from 12 subjects per group (see section 3.3).

As an alternative to subject-specific models, group-average models that represent groups of similar age and gender (e.g. 14–17 year old males, 55–80 year old females, etc.) can be developed to investigate and predict differences in TBI risk. In this case, imaging data from the subjects within a particular group must be co-registered to some common space, which presents several challenges (see section 3.2). However, this approach offers opportunities for understanding the range of expected observations in a group and providing confidence intervals on predictions. This insight can similarly be achieved through an ensemble of subject-specific models that represent the range of anatomy and physiology in the general population and in which the accuracy of each model can be quantified. Such an ensemble of models would provide insight into the range of outcomes of a typical impact, and could identify common features of brain mechanics that might inform strategies for preventing or treating TBI. Both approaches (group-average models and ensembles of subject-specific models) require many experimental data sets across the population, which is a major contribution of this study.

### 3.2. Opportunities and Challenges with Incorporating Imaging Data in Models

While the imaging data collected in the study offer a wealth of information to modelers, there are multiple challenges when using imaging data in creating models. Many models use discrete regions based on anatomical image segmentations of white and gray matter, ventricles, and the falx and tentorium; however, such segmentations often impart sharp boundaries in the model or have to be smoothed to generate a realistic surface. Additionally, many regions important for brain biomechanics such as the cortical cerebrospinal fluid, meninges, and skull are not easily distinguished and require refined algorithms to segment automatically. The issue of discrete region segmentation is particularly important in building group-averaged models as the imaging data from multiple subjects must be co-registered to create necessary inputs. Registration strategies should be considered carefully, and ideally the error and uncertainty involved in these steps should be quantified.

The MRE data collected in this study offers an opportunity to also incorporate spatial heterogeneity in mechanical properties through detailed maps of shear modulus, which exhibit considerable variability between regions and across subjects^47^. However, it is important to note that while MRE measurements can provide linear viscoelastic material properties that govern very small shear deformations to the brain (on the order of ~10^−4^–10^−3^ strain)^86^, realistic head injury scenarios involve much larger strain magnitudes (~10^−1^–10^0^ strain)^54,101,123^. Thus, MRE data do not capture stress-strain nonlinearity or features of finite strain behavior such as strain stiffening and compression-tension asymmetry. In contrast, many 3D brain simulations have implemented visco-hyperelastic material models^15,38,69,79,94^, with quasi-linear viscoelasticity using a Prony-series formulation of the relaxation modulus to describe behavior at dynamic strain rates^31,123^. Care and insight are required in using MRE data to parameterize the material models used for studying brain injury. Possible strategies include combining the spatial heterogeneity and magnitudes of shear moduli from MRE at small strains (<0.01 strain), with data from tagged MRI (0.05–0.10 strain), and data from large-strain responses in *ex vivo* tissue. Some researchers have already begun incorporating the MRE data in their models^37,70^, yet the optimal approach to this problem remains an open challenge.

Quantifying the uncertainty and accuracy of models also requires consideration. Each choice in creating a computational model from experimental data involves approximations or assumptions that impart uncertainty to the model. Quantifying this net uncertainty and its effect on the model predictions is desirable for a fair and rigorous comparison of model predictions to experimental observations (which have their own uncertainties). A typical uncertainty quantification (UQ) framework for computational models comprises^108^: (i) modeling the system, (ii) defining probabilistic models of input parameters to quantify the sources of uncertainty, and (iii) propagating the uncertainties from the input parameters through the computational model.

Monte Carlo (MC) simulations are a highly versatile and popular approach for propagating uncertainties through computational models, though computationally expensive^13^. For complex systems such as 3D models of brain biomechanics, surrogate models (also called metamodels) offer an efficient and powerful tool for UQ, as they approximate the behavior of a computational model with negligible computational cost per run. In addition to UQ, these models can also be used to conduct sensitivity analyses to identify the key parameters that have the strongest influence on the model predictions. Such information is helpful for both modelers (as it identifies “important” and “unimportant” input parameters) and experimentalists (as it identifies the measurements in which uncertainty should be minimized). Specific objectives in this area include developing agreement on metrics relevant to brain mechanics and TBI, and identifying metadata that captures uncertainties in both experiments and simulations.

A particular challenge in evaluating the accuracy of predicted 3D displacement or strain fields from computational models is defining objective metrics that capture the most important features of the prediction. Some simulation studies focus on prediction of injury from global, scalar quantities such as the maximum principal strain or the fraction of elements exceeding a certain threshold^30,89^; if these global scalar quantities are used for model evaluation, it discounts much of the time-resolved, tensorial field data available. Metrics proposed for model evaluation include the Correlation Score (CS)^59^, Correlation and Analysis (CORA)^33^ metric, ISO score^8^, and other generic error metrics such as root mean square error (RMSE) or sum of squared error (SSE). Such scores are helpful in summarizing the relationships between dynamic 3D data sets, typically using one number that indicates a benchmark level of accuracy. However, there does not yet appear to be consensus on how these scores can be used for calibration or evaluation^124^. In part this is because of the tensorial nature of the key fields: deformation and strain, along with their temporal variations. To that end, there have been efforts to recalibrate^35^ or design anew other metrics that measure spatiotemporal similarity of a tensor field^42^. Given the evolution of imaging methods that provide the full strain tensor as a function of position (even if at lower spatial or temporal resolution), we advocate the comparison of deformation (strain) predictions from a simulation with all of the available experimental strain data from corresponding loading conditions, including their variations across time. Continuing investigations into evaluation metrics and their interpretation, supplemented with the increased availability of brain deformation data, will allow for objective and unbiased evaluation of the next generation of computational brain models.

This project will provide data on brain anatomy and material properties from individuals grouped by age and sex, with the goal of allowing for the development of group-average models. The target for this project is to have such data from at least 12 individuals within each group; this number is a feasible contribution within the project period, but it is not known *a priori* whether it is a sufficient number for robust statistical characterization of the population at large. Challenges remain for the modeling community in how to combine these anatomical and physical data sets into useful models. For example, it is not obvious what is the best way to construct a mechanically-representative, group-average geometry from a set of anatomical images. The variability in anatomy and properties within each group, however, might inform confidence intervals for model parameters and predictions.

### 3.3. Limitations

As noted above, although all subjects in these studies will undergo multiple types of imaging scans, only a small subset of the subjects (< 5 subjects) will undergo *every* type of scan; thus, only a few of the subject-specific models can incorporate *all* classes of imaging data (subject specific anatomy, microstructure, and material properties). These few models will be valuable to compare with models that are subject-specific in terms of anatomy and microstructure, but with group-averaged material properties.

The sub-injury levels of skull acceleration in these studies mean that observations apply only indirectly to injurious events, thus a computational model that is calibrated solely using *in vivo* tagged MRI data for predicting injury may have significant uncertainty in predicting the response to higher accelerations. Existing computational models of the human brain have been evaluated using both high-rate cadaveric impact data^46^ and *in vivo* head rotation data^62^. While the former is generally associated with large peak acceleration amplitudes (≿ 8000 rad/s^2^) and small impulse durations (~3–5 ms), the latter consists of much smaller peak accelerations (< 500 rad/s^2^) and longer impulse durations (~40 ms). Importantly, many of the real-life concussive and subconcussive head impacts involve peak accelerations and loading durations that lie between these two extremes. This highlights the importance of considering both high-amplitude cadaveric and low-amplitude *in vivo* (tagged MRI) datasets for model evaluation^123^. Similarly, a model built and calibrated using only cadaveric data may be poor at predicting the response of the brain *in vivo*. Opportunities exist to bridge this gap by using both experimental imaging data collected in the living brain, and data from the cadaveric brain collected under higher accelerations and shorter loading durations. Complementary animal studies, for example in the pig, might be used to compare the response of *in vivo* and *ex vivo* brain behavior, although comparisons must account for the differences in size and anatomy.

MRE data are obtained under small strain conditions and thus do not directly reflect large-strain behavior important for modeling injury. Additionally, in MRE the assumption is typically made that brain tissue is isotropic, to simplify the property estimation problem. This assumption ignores the mechanical anisotropy imparted by the organization of axons in white matter tracts^25,26,92,115^. Anisotropy of white matter can be incorporated in the constitutive model^18,31,34^ or modeled explicitly,^32,117^ including the spatially varying direction of fiber alignment and the degree of dispersion both of which can be measured using DTI. Currently, assumptions about mechanical anisotropy are supported only by very limited data. While the degree of mechanical anisotropy in white matter is still a topic of active research^15^, anisotropic MRE of the brain is currently under development by our group and others^73,93,114^, with preliminary estimates of shear and tensile anisotropy of at least 10–30% in the fiber direction in white matter tracts^104,107^.

Practical considerations like MRI scan time and scanner coil size limit the type, duration, and frequency of loading for data collected in this project. All these aspects remain open areas of development for our group. In future work we anticipate broadening the types of motion and range of impact duration in tagged MRI experiments, and the harmonic excitation frequencies used in the MRE experiments.

## 4. Outlook

Through the data collected, processed, and disseminated in this project, we aim to provide new resources for developing computational brain models. Using MRI techniques, full field measurements of displacement, strain, and mechanical properties throughout the brain, acquired at sub-injury levels of skull acceleration, are now available in male and female subjects of ages from adolescence through older adulthood, with more data to be released as it is collected over the next several years. These data complement traditional biomechanics measurements from animal studies and cadaveric specimens, and they provide new avenues for parameterizing and evaluating models of brain biomechanics. With the range of participants involved, we expect models will be able to account for differences between individuals and investigate the potential effects of age and sex on the biomechanical response of the brain and risk of injury.

We look forward to working with the modeling community to incorporate these data, along with other brain biomechanics measurements from animal models and cadaveric specimens, into the next generation of brain biomechanics models. We believe this approach will be synergistic, enhancing the utility of both (i) the current, MRI-derived data from human volunteers, and (ii) complementary cadaveric data from other studies, for modeling the response of the intact, living human brain to injury-level accelerations. We hope these studies stimulate future interactions with and among those who develop and use computational models of brain biomechanics.

## Data Availability

All data described in this work is available at http://www.nitrc.org/projects/bbir.
